# A Novel Standardized Method for Aiding to Determine Left Atrial Enlargement on Lateral Thoracic Radiographs in Dogs

**DOI:** 10.3390/ani13132178

**Published:** 2023-07-02

**Authors:** Viktor Szatmári, Zelie M. M. Hofman, Nynke J. van Bijsterveldt, Anna R. Tellegen, Federico R. Vilaplana Grosso

**Affiliations:** Department of Clinical Sciences, Faculty of Veterinary Medicine, Utrecht University, Yalelaan 108, 3584 CM Utrecht, The Netherlands

**Keywords:** dyspnea, heart failure, myxomatous mitral valve degeneration, pimobendan, stage B

## Abstract

**Simple Summary:**

Measuring left atrial size has important prognostic and therapeutic consequences in cardiac diseases. An enlarged left atrium indicates that the cardiac disease is severe. Though cardiac ultrasound examination is the best way to determine left atrial size, this technique is often unavailable in veterinary practices, as it is expensive and requires specific expertise. Therefore, chest X-rays are usually used to decide whether the left atrium is enlarged. With that, practicing veterinarians aim to differentiate cardiac from respiratory diseases, which both can lead to similar clinical signs. However, determining left atrial enlargement on X-rays can be challenging. The authors of this study came up with a simple and reproducible method that might make determining left atrial size on X-rays easier. The study aimed to compare two methods. Dogs with and without cardiac disease were included. The left atrial size of the included dogs had also been determined with ultrasonography to serve as a reference. First, 39 veterinarians and veterinary students interpreted 90 canine chest X-rays subjectively. At least two weeks later, the same observers applied the new method on the same radiographs. The new standardized method did not give a more accurate diagnosis than subjective assessment.

**Abstract:**

Background: Left atrial enlargement indicates severe cardiac disease. Although the gold standard for determining left atrial size is echocardiography, many veterinary practices lack the necessary equipment and expertise. Therefore, thoracic radiography is often used to differentiate cardiogenic pulmonary edema from primary respiratory diseases and to facilitate distinguishing dogs with stage B1 and B2 mitral valve degeneration. Methods: The goal was to test a new standardized method for identifying radiographic left atrial enlargement. On a lateral radiograph, a straight line was drawn from the dorsal border of the tracheal bifurcation to the crossing point of the dorsal border of the caudal vena cava and the most cranial crus of the diaphragm. If a part of the left atrium extended this line dorsally, it was considered enlarged. Echocardiographic left atrial to aortic ratio (LA:Ao) was used as a reference. Thirty-nine observers with various levels of experience evaluated 90 radiographs, first subjectively, then applying the new method. Results: The new method moderately correlated with LA:Ao (r = 0.56–0.66) in all groups. The diagnostic accuracy (72–74%) of the subjective assessment and the new method showed no difference. Conclusions: Though the new method was not superior to subjective assessment, it may facilitate learning and subjective interpretation.

## 1. Introduction

Cardiac diseases are frequently diagnosed conditions in dogs. Most of these diseases cause left-sided volume overload, with myxomatous mitral valve degeneration (MMVD) and dilated cardiomyopathy being the most common acquired diseases and patent ductus arteriosus (PDA) and ventricular septal defect being the most common congenital cardiac diseases [1,2]. Chronic severe left ventricular volume overload inevitably leads to increased left atrial (LA) pressure and LA dilatation [1,2,3]. In a subset of dogs, the increased LA pressure eventually leads to the development of cardiogenic pulmonary edema due to left-sided congestive heart failure (L-CHF) [1,4]. Clinical signs of L-CHF, such as dyspnea, exercise intolerance, and cough, can be similar to those of primary chronic respiratory diseases, such as trachea-bronchomalacia and chronic bronchitis [1,4]. Because both prognosis and therapy for primary respiratory disorders and L-CHF differ, an accurate diagnosis is essential. 

Thoracic radiography is a routinely chosen first-diagnostic test following physical examination in dogs with respiratory signs to evaluate the pulmonary and cardiovascular structures [1,5,6,7]. Left atrial enlargement, as a manifestation of increased LA and pulmonary wedge pressures, is present in any chronic left-sided cardiac disease before cardiogenic pulmonary edema arises [1,5]. Therefore, diagnosing LA enlargement facilitates differentiation between L-CHF and a primary respiratory condition in a dyspneic dog with radiographic pulmonary infiltrates [1,4,5]. By establishing LA enlargement, the severity and stage of certain cardiac diseases in asymptomatic dogs can also be assessed [1,7,8]. In subclinical MMVD (stage B), staging has important prognostic and therapeutic consequences [1,7,8]. Stage B1 identifies dogs with mild disease, with normal LA and left ventricular chamber size, while stage B2 identifies dogs with more advanced disease, with left ventricular and LA enlargement [1,8]. Though for stage B1 currently no effective therapy is known, initiating lifelong medical therapy with pimobendan in dogs in stage B2 MMVD has been shown to delay the onset of L-CHF and prolong longevity [8]. 

The standard method to determine LA size in dogs is two-dimensional echocardiography [1,3]. The most widely used technique is to measure the LA to aortic ratio (LA:Ao), which is a body weight-independent linear variable and should be under 1.7 in healthy dogs [3]. However, due to the limited availability of ultrasound equipment and the expertise needed for acquiring and interpreting echocardiographic images, this is not widely available in general veterinary practices [8,9,10]. 

Radiography, on the other hand, is available in most first-opinion veterinary practices, and it is also the gold standard for confirming cardiogenic pulmonary edema [1,4,5,10]. Thoracic radiographs in dogs can provide information not only on the lungs and the size of the cardiac silhouette but also on the LA size [5,7]. However, the interpretation of thoracic radiographs is often subjective and sensitive to errors, especially if performed by non-radiologists, such as first-opinion veterinary practitioners [5,7]. In order to improve the diagnostic value of radiographs for assessing cardiomegaly, vertebral heart score (VHS) was introduced several decades ago and has been used widely in practice ever since to provide a standardized and reproducible measurement for veterinary practitioners [6,8]. Because VHS evaluates the cardiac silhouette as a whole, pericardial effusion and right-sided heart diseases (e.g., resulting from severe tricuspid valve regurgitation) can also lead to high values. For determining LA enlargement on radiographs, several subjective and standardized methods have been described utilizing either dorsoventral or lateral radiographs: the tracheal bifurcation angle [11], the radiographic left atrial dimension (RLAD) [12], the vertebral left atrial size (VLAS) [13], and the modified vertebral left atrial size (M-VLAS) [14]. Subjective signs of LA dilatation on lateral radiographs include the appearance of a dorso-caudally located soft tissue bulge of the cardiac silhouette (so-called “tent”), straightening of the caudal margin of the cardiac silhouette (i.e., disappearance of the waist), and dorsal displacement of the trachea, left principal bronchus or caudal vena cava [12,15]. 

For practicing veterinarians and for teaching purposes, there is a need for a simple and reproducible method to reliably differentiate normal from markedly enlarged LA on radiographs when echocardiography is unavailable [9,15,16,17,18,19,20,21]. In the present study, the authors created and subsequently tested a new method, the so-called “crossing lines method”, to assess LA size on lateral thoracic radiographs in dogs with and without LA enlargement. The idea of this method is based on the specific assessment of the caudo-dorsal bulge on the cardiac silhouette on lateral radiographs in dogs with marked LA dilatation. The aims of the present study were (1) to compare the accuracy of the “crossing lines method” with a subjective assessment of LA size (normal vs. enlarged) on lateral radiographs and (2) to determine how closely the “crossing lines method” correlates with the echocardiographic LA:Ao. The null hypotheses were that the “crossing lines method” would not correlate with echocardiographic LA:Ao and that the “crossing lines method” would not be superior to subjective assessment used by inexperienced observers.

## 2. Materials and Methods

### 2.1. Study Sample

Patient records of dogs were searched using the clinic’s electronic database, which underwent an echocardiographic examination and thoracic radiographs at the authors’ veterinary teaching hospital on the same day in a period of five years. Cases were included only if the echocardiography was performed by either a board-certified veterinary cardiologist (ECVIM-CA) or an ECVIM-CA cardiology resident under the direct supervision of a cardiology specialist. Radiographs, where a cardiac implant was seen, such as a pacemaker or an occlusion device for PDA, were not included because this would cause interpretation bias of having a cardiac disease. In addition, radiographs, where the cardiac silhouette was obscured by dense pulmonary infiltrates or pleural effusion, were not included either.

Based on the echocardiographic LA:Ao, the case was allocated either to the group of normal-sized LA with a LA:Ao < 1.70, or to the group of enlarged LA when LA:Ao ≥ 1.70 [3]. The images for LA:Ao were obtained on unsedated, manually restrained dogs in right lateral recumbency, using two-dimensional grey-scale images from the standard right parasternal trans-aortic short axis view [3]. The LA:Ao ratio was measured on the first measurable frame after aortic valve closure [3].

### 2.2. Observers

There were three groups of observers who tested the “crossing lines method”. They all participated voluntarily. The least experienced group consisted of twenty-one fourth-year veterinary students, who had not followed any radiology courses yet, and therefore had no expertise in interpreting radiographs. The more experienced group consisted of ten sixth-year (final year) veterinary students who had already followed a course on interpreting radiographs. The most experienced group consisted of eight first-opinion veterinary practitioners who had been working as practicing veterinarians for a median of three years (range 2–14 years) prior to participating in the study. The new method was tested on the most experienced group first and the least experienced last.

Furthermore, four board-certified veterinary radiologists (ECVDI) evaluated the radiographs of the study sample. These four participants used their subjective assessment only, not the “crossing line method”. The reason for adding this group was to assess whether determining LA enlargement on radiographs was feasible at all in the set of selected radiographs.

All observers were blinded to the clinical and echocardiographic findings and the radiographic assessment of the other participants.

### 2.3. The New “Crossing Lines Method”

**Figure 1:**
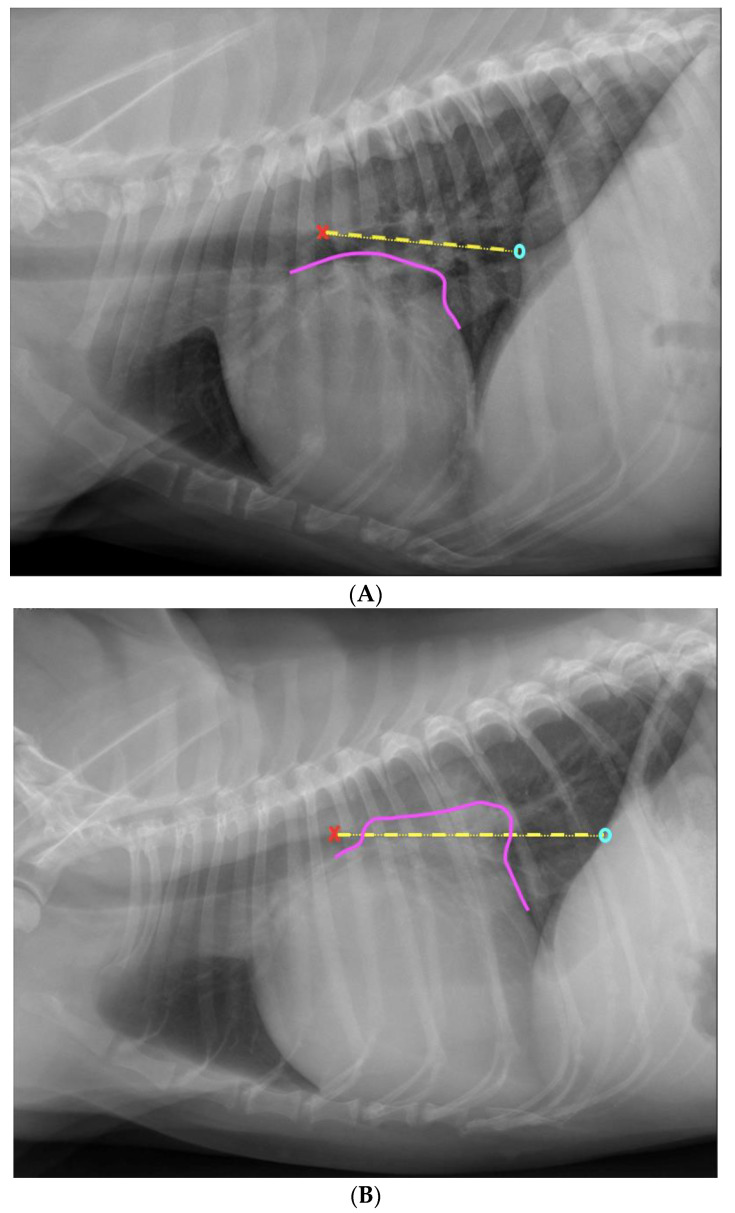
Left lateral thoracic radiographs of two dogs. The dorsal border of the tracheal bifurcation is indicated with a red “x”. The crossing point of the dorsal border of the caudal vena cava and the most cranial crus of the diaphragm is shown as a light blue “0”. The straight line between these two points is shown as a yellow interrupted line. (**A**) Applying the “crossing lines method” in a dog with a normal-sized left atrium. The dorsal border of the left atrium is shown with a magenta line, which does not cross the yellow interrupted line; therefore, the left atrium is considered normal in size. (**B**) Applying the “crossing lines method” in a dog with a markedly enlarged left atrium. The dorsal border of the left atrium is shown with a magenta line, which crosses the yellow interrupted line. Therefore, the left atrium is considered enlarged.

The “crossing lines method” was obtained by drawing a straight line from the dorsal border of the tracheal bifurcation to the crossing point of the dorsal border of the caudal vena cava and the cranial crus of the diaphragm on a left lateral radiograph. If a part of the cardiac silhouette extended beyond this line dorsally, the LA was judged to be enlarged (Figure 1). According to clinic standards, lateral radiographs were routinely taken in left lateral recumbency at the authors’ institution.

### 2.4. Study Protocol

All participants who tested the “crossing lines method” assessed the same set of radiographs twice, using two different methods. During the first assessment, the participants assessed the LA size on lateral thoracic radiographs subjectively and recorded their results as a 0 (LA is normal) or 1 (LA is enlarged) on a form. Before the assessment, all participants were provided with written instructions (two pages) and an example of a canine lateral thoracic radiograph with a normal-sized LA and one with a markedly enlarged LA, together with explanations. The observers were not instructed, other than these two example radiographs provided, how to assess the LA size subjectively. Anatomical structures and the silhouette of the LA were marked on the radiographs. In the written part of the instruction, the caudo-dorsal bulge was described as a typical sign of LA enlargement.

In addition, the fourth-year students were asked to measure the VHS too on the same radiographs. These fourth-year students were also provided with a separate instruction sheet on how to measure the VHS. The reason for asking the least experienced group to perform VHS measurements was to reveal whether performing measurements on radiographs was a reasonable expectation at all.

During the second assessment, the same radiographs were shown to the same participants, but in a different order, and there was at least a two-week time interval between the two assessments so that the participants would not remember specific radiographs. The participants could determine when to evaluate the images for the second time as long as it was beyond 14 days after the first assessment. At the second assessment, the participants were asked to assess the LA size on lateral radiographs using the “crossing lines method” and record their findings as a 0 (LA is normal) or 1 (LA is enlarged) on a form. The participants assessed the radiographs on a computer screen using viewing software (Enterprise Imaging XERO viewer 8.1.2., AGFA HealthCare N.V., Mortsel, Belgium). All participants were provided with written instructions (two pages), including four images, two without and two with annotations on how to apply the “crossing lines method” (Figure 1).

The specialists in veterinary diagnostic imaging assessed the LA size on the same radiographs subjectively. This group had access to all available radiographs of each case, including a dorsoventral view and a second lateral view if it was made. They marked the cases with a 0 (LA is normal sized) or 1 (LA is enlarged) on a form, too. The radiologists did not apply the “crossing lines method”. The reason for this study design was that it would not have been realistic to expect from the radiologists to put their expertise aside and interpret the radiographs solely using the novel “crossing lines method”. If we had done this, it would have caused biased results. The “gold standard” for radiographic assessment of LA size in this study was considered the subjective interpretation performed by the radiologists.

Lastly, a resident in veterinary diagnostic imaging measured the VHS on all lateral radiographs. This set of data served as a reference from an expert to compare the values measured by the fourth-year students.

### 2.5. Data Analysis

For the statistical analysis, commercially available software (SPSS Statistics for Macintosh, Version 27.0. IBM Corp., Armonk, NY, USA) was used.

Descriptive statistics were generated. To determine whether the data were distributed normally, the Kolmogorov–Smirnov test was used. 

To reveal whether the study sample had a wide range in LA sizes, the Mann–Whitney test was used. 

To investigate if there was a correlation between the echocardiographic LA:Ao and the subjective LA size assessed on radiographs by ECVDI specialists, Spearman’s rho was used. Crosstabs were also made to assess the percentages of correct diagnoses. To evaluate if there was a correlation between the echocardiographic LA:Ao and the “crossing lines method” for the different observer groups, Spearman’s rho was used. Receiver operating characteristic curves (ROC) were constructed for the results of the radiologists and the sixth-year students to reveal the performance of the test.

To reveal whether the “crossing lines method” was superior to the subjective assessment of the LA size on lateral radiographs and as accurate as the echocardiographic LA:Ao, the results obtained from the “crossing lines method” and those from the subjective assessment were compared to each other and also to the echocardiographic LA:Ao. To compare these data, cross tabs were made. For the comparison of the subjective assessment and the “crossing lines method”, the mean scores of the various observer groups were compared using the non-parametric Wilcoxon signed-ranks test. This was done separately for cases with a normal LA and cases with an enlarged LA. Crosstabs were also made to assess the sensitivity, specificity, and accuracy of each observer group.

To investigate whether experience played a role when using the subjective assessment and the “crossing lines method”, the means of the subjective assessments and those of the “crossing lines method” of each observer group were compared using the non-parametric Wilcoxon signed-ranks test. 

Spearman’s rho and ANOVA were used to determine whether there was a correlation between (1) the subjective assessment of LA size determined by the fourth-year students and the echocardiographic LA:Ao, and (2) the VHS measured by the fourth-year students and by the ECVDI-resident. The strength of correlation was classified as follows: 0.91–1.00 very strong, 0.71–0.90 strong, 0.51–0.70 moderate, 0.31–0.50 weak, and 0.01–0.30 very weak correlation.

The *p*-values less than 0.05 were considered statistically significant.

## 3. Results

### 3.1. Time Interval between the Two Assessments

Between the two assessments of the fourth-year students, there was a median of 30 days (range 22–59 days). Two of the twenty-one participants submitted an incomplete dataset; one assessed only 10 cases for the VHS, and the other one did not perform an assessment with the “crossing lines method” at all. Data from the incomplete forms were not analyzed. Between the two assessments of the sixth-year students, there was a median of 26 days (range of 15–69 days). Between the two assessments of the practicing veterinarians, there was a median of 49 days (range of 27–76 days).

### 3.2. Echocardiographic Diagnoses

Radiographs of 90 dogs were included. Echocardiography revealed an enlarged LA in 48 cases and normal-sized LA in the remaining 42 dogs. The LA:Ao ratio data were not normally distributed (*p* = 0.000). The median LA:Ao for the normal-sized LA was 1.30 (range 0.96–1.60), and the median LA:Ao for the enlarged LA was 2.18 (range 1.70–4.30). The echocardiographic diagnosis of the dogs is shown in Table 1.

### 3.3. Dog Breeds Included

A large variety, namely 40 different breeds, was represented in the study. The three most common breeds were: mixed breed (*n* = 13), (Cavalier) King Charles spaniel (*n* = 12), and Labrador retriever (*n* = 7).

Among the 16 dogs with structurally normal hearts, 6 were mixed breeds, and 2 were Bouvier des Flandres; the rest were single breeds. Only one of the dogs with a structurally normal heart, an American bulldog, had an increased LA:Ao (1.8).

Among the 34 dogs with MMVD, 10 were Cavalier King Charles spaniel, 3 were Dachshound, 2 were Cairn terrier, 2 were Jack Russell terrier, and the rest were single breeds. Nine of the dogs with MMVD were in stage B1, and the remaining twenty-five dogs were either in stage B2 or C.

In the group of dogs with dilated cardiomyopathy, only Flatcoated retrievers were represented with more than one individual, namely three. Six of the nineteen dogs with dilated cardiomyopathy had LA enlargement.

Among the dogs with mitral valve dysplasia, bull terriers were overrepresented with four individuals; three of the six dogs had LA enlargement.

Of the five dogs with PDA, one had right-to-left, and four had left-to-right shunting. Sarloos wolfhounds were overrepresented, with two individuals, in the PDA group. Two dogs with PDA had LA enlargement, of which one dog was in L-CHF.

In the group of pulmonic stenosis, two of the three dogs were French bulldogs.

All other conditions were diagnosed only in single breeds. All dogs with the diagnosis of aortic stenosis, pulmonic stenosis, pulmonary hypertension, pericardial effusion, and tachycardia-induced cardiomyopathy had normal-sized LA.

### 3.4. Correlation between the Echocardiographically and Radiographically Assessed Left Atrial Size

**Figure 2:**
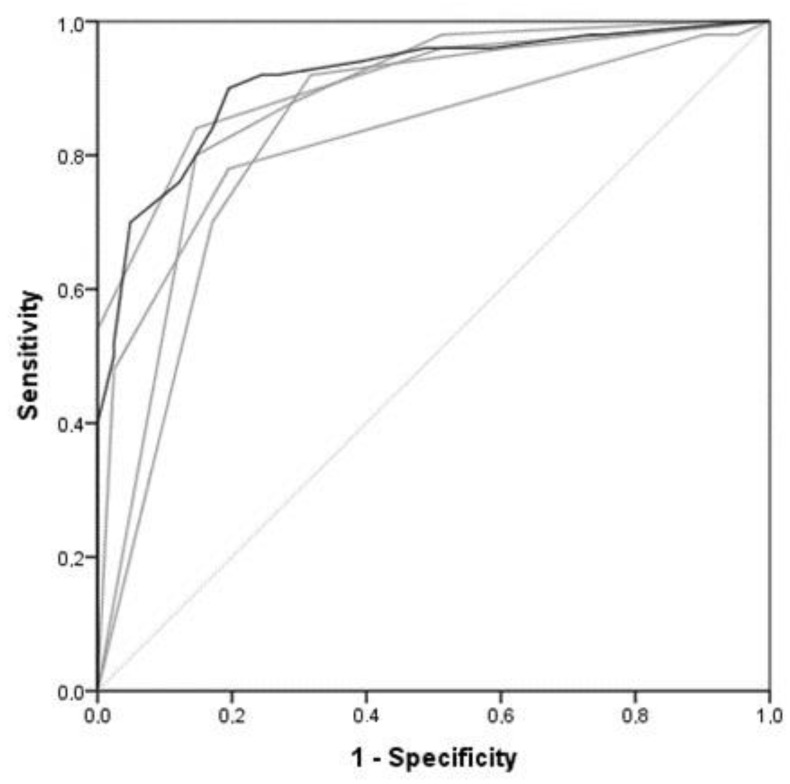
Receiver-operating characteristic curves (ROC) show the four radiologists’ subjective assessment in determining left atrial size on thoracic radiographs. Each grey line represents the results of an individual radiologist, while the darker line shows the combined results of the four radiologists. The lightest grey line is the reference line. The mean area under the curve (AUC) was 0.91, SE 0.03 (*p* < 0.001), 95% C.I. [0.86–0.97].

To assess the radiologists’ performance in the subjective assessment of LA size on radiographs, ROC curves were generated. The radiologists’ results were used as the gold standard, reflecting the highest possible achievable accuracy for this study. There was a positive correlation between the echocardiographic LA:Ao and the subjective assessment of LA size on thoracic radiographs performed by four ECVDI specialists (r = 0.654, *p* < 0.001). Their accuracy was 81% (Table 2, Figure 2).

**Figure 3:**
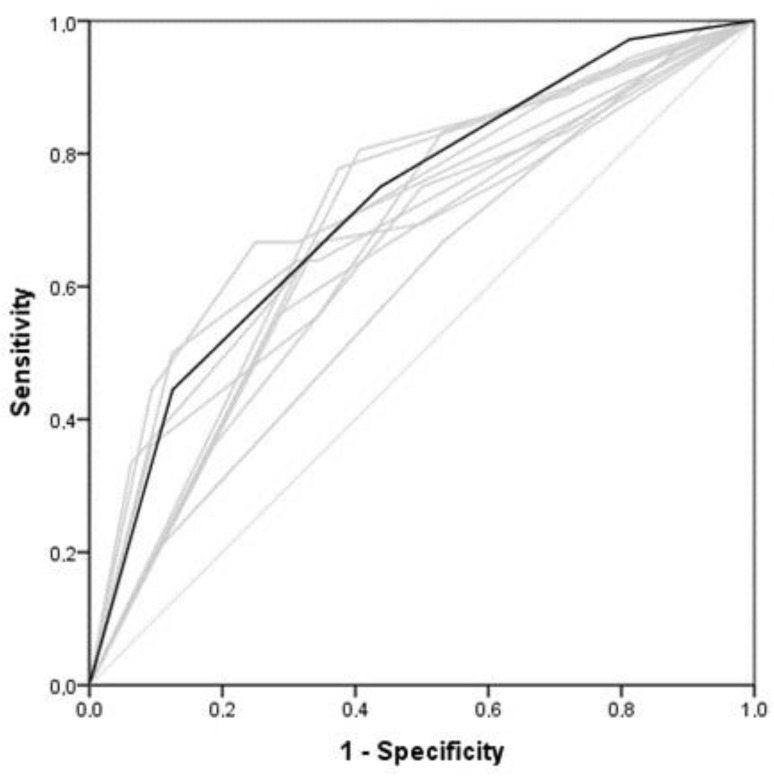
Receiver-operating characteristic curves (ROC) show the assessments of ten sixth-year students in determining left atrial size on thoracic radiographs using the “crossing lines method”. Each grey line represents the results of an individual student, while the dark line shows the combined results of the ten students. The lightest grey line is the reference line. The mean area under the curve (AUC) was 0.72, SE 0.06 (*p* < 0.01), 95% C.I. [0.60–0.84].

To assess the performance of the “crossing lines” method applied by inexperienced observers, ROC curves were generated. There was a moderate correlation between the echocardiographic LA:Ao and the “crossing lines method” used by the fourth-year students (r = 0.56), the sixth-year students (r = 0.55) (Figure 3), and the practicing veterinarians (r = 0.66) (*p* < 0.001).

### 3.5. Comparing Subjective Assessment with the “Crossing Lines Method” for Determination of Left Atrial Enlargement

The fourth-year students reached a correct diagnosis in 72% of the cases when they subjectively assessed the radiographs and in 70% of the cases when they applied the “crossing lines method”. The subjective assessment was more accurate than the “crossing lines method” to diagnose a normal-sized LA (*p* = 0.034). When the LA was enlarged, the performance of the subjective assessment and the “crossing lines method” did not differ (Table 3).

The sixth-year students reached a correct diagnosis in 72% of the cases when they subjectively assessed the radiographs and in 71% of the cases when they applied the “crossing lines method” (Table 4).

Practicing veterinarians reached a correct diagnosis in 77% of the cases when subjectively assessing the radiographs and in 75% of the cases when they applied the “crossing lines method” (Table 5).

The sensitivity was significantly higher in the group of practicing veterinarians compared to the results of both student groups, regardless of the method used. The sensitivity of subjective assessment was significantly higher when comparing all the students with the practicing veterinarians. When looking at the “crossing lines method”, the sensitivity was only significantly different between the fourth-year students and the practicing veterinarians. Regarding sensitivity, specificity, and accuracy, there were no significant differences between the subjective assessment and “crossing lines method” for each group (Table 6).

### 3.6. Effect of Experience

To determine if experience is an important factor when using the subjective assessment to determine LA size on lateral thoracic radiographs, the means of the observers were compared. The fourth-year students scored better in recognizing normal-sized LA than the sixth-year students (*p* = 0.048). Practicing veterinarians were able to recognize LA enlargement better than both fourth-year students (*p* = 0.044) and sixth-year students (*p* = 0.027) (Table 7).

To determine if the experience was an important factor when using the “crossing lines method”, the means of the observers were compared. Practicing veterinarians detected an enlarged LA more accurately than fourth-year students (*p* = 0.045) (Table 8).

### 3.7. Correlation between Vertebral Heart Scale (VHS) and Left Atrial Size

**Figure 4:**
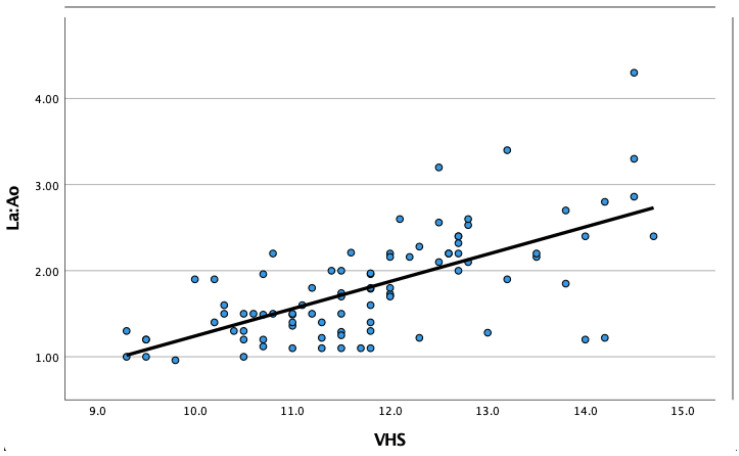
This scatter plot graph shows a moderate positive correlation between the echocardiographic left atrial to aortic ratio (La:Ao) measured by a specialist or resident in veterinary cardiology and the vertebral heart scale (VHS) measured on lateral thoracic radiographs by a resident in veterinary diagnostic imaging in 90 dogs.

In order to determine if students consciously or unconsciously correlated an enlarged cardiac silhouette with an enlarged LA on the lateral radiographs when assessing the LA size subjectively, the correlation between the VHS and their score of the subjectively assessed LA was compared. There was a positive correlation (r = 0.511, *p* < 0.001) between VHS and the LA size when the mean VHS and the mean LA size were subjectively determined for each case by the fourth-year students. However, there was also a positive correlation (r = 0.667, *p* < 0.001) between the VHS measured by the resident in veterinary diagnostic imaging and the echocardiographic LA:Ao (Figure 4).

Comparing the VHS measured by the resident in veterinary diagnostic imaging and the VHS measured by the fourth-year students showed a very strong positive correlation (r = 0.97, *p* < 0.001).

## 4. Discussion

The “crossing lines method” is historically the first reported standardized technique that attempted to objectively determine LA enlargement on lateral thoracic radiographs in dogs and dates back to 2016 [21]. Some years after its initial presentation as an abstract at an international congress, several new methods have been published with the same aim [8,9,10]. 

In the present study, the tested new “crossing lines method” did not perform better than subjective assessments of LA size on lateral radiographs when it was applied by observers of various levels of experience in interpreting thoracic radiographs. Neither method (subjective or the “crossing lines”) and none of the groups succeeded in reaching the accuracy of subjective interpretation of radiologists or that of the echocardiographic LA:Ao. Therefore, the null hypotheses of the study cannot be rejected with strength. Though it is unknown which subjective criteria the inexperienced participants used when they assessed the LA on lateral radiographs, it is plausible that they focused on the dorso-caudal bulge, as this one was mentioned in their instruction sheet. The other criteria are either less specific, as the dorsal displacement of the trachea would also be present with generalized cardiomegaly, without LA dilatation, or more difficult to appreciate, as straightening of the caudal margin of the cardiac silhouette. These considerations could explain why no statistically significant difference was found between the subjective assessment of LA size and the application of the “crossing lines method”, as the participants might have used similar criteria with both methods. The LA is a complex three-dimensional structure, which is hard to evaluate with a single line on a two-dimensional image. On lateral thoracic radiographs, the various reported methods chose to investigate either the cranio-caudal length (VLAS) or its oblique dimension (RLAD). The “crossing lines method” aimed to investigate the ventro-dorsal length of an enlarged LA. In theory, all these methods should be able to differentiate a normal-sized LA from a markedly enlarged one as LA enlargement causes a caudo-dorsal buldge on the cardiac silhouette.

Certain breeds, such as Beagle, Boxer, and English setter, have reportedly higher LA:Ao than the recommended cut-off of 1.7 [3]. Because our study consisted of only a single dog of these breeds, namely a Boxer with a LA:Ao of 1.49, the study results were not influenced by this. However, there was an American bulldog without structural heart disease whose LA:Ao was abnormally high, assumably because of breed-related aortic hypoplasia. Nevertheless, chest confirmation might influence the radiographic appearance of an enlarged LA. Similarly, breed-specific reference values are established for VHS.

Not surprisingly, experience and training turned out to be an important factor in interpreting radiographs in our study, as the accuracy increased with experience. However, the “crossing lines method” was less affected by this factor than the subjective assessment. Therefore, the “crossing lines method” might have advantages in teaching and in the hands of less experienced observers. Though experience might increase interobserver agreement, a study showed that the accuracy in subjective assessment of radiographic cardiomegaly did not increase with experience; only the level of confidence did [7,15]. 

In our study, the fourth-year students did not seem to make a conscious or unconscious correlation between cardiomegaly (i.e., VHS) and the subjectively assessed LA size. Interestingly, the positive correlation between these variables was higher when these two variables were assessed by the resident in diagnostic imaging (r = 0.67) as compared to the fourth-year students’ assessments (r = 0.51). The most likely reason for this finding is that most dogs with cardiomegaly had an underlying disease that led to both LA and left ventricular dilation.

Regarding interobserver variability of VHS measurement, there was a strong correlation when measured by the fourth-year students and the resident, showing that VHS is a reliable tool even in the hands of untrained students. This means that the students had no difficulties in identifying the reference points, so applying the “crossing lines method” by this group should be feasible, too.

There are several methods in the veterinary literature that were developed for the same purpose as the “crossing lines method”, but all have their own shortcomings [11,12,13,14,15,16,17,18,19,20]. The oldest method, published in 2012, measures the tracheal bifurcation angle on a dorsoventral radiograph, based on the principle that LA enlargement would cause more deviation, therefore, a larger angle of the principal bronchi [11]. On a dorsoventral radiograph, the LA is located in the midline between the principal bronchi. The tracheal bifurcation angle describes the angle formed by the two principal bronchi, which range between 60 and 90 degrees in healthy dogs. A wider angle could be the result not only of LA enlargement but also of tracheobronchial lymphadenopathy. Unfortunately, this technique had a very low sensitivity. 

Another method, described in 2018, is the radiographic LA dimension (RLAD) [12]. For this, the VHS needs to be measured first. Afterwards, a straight line is drawn from the dorsal edge of the LA to the crossing point of the lines used to determine the VHS. Subsequently, this line is positioned along the vertebral bodies starting from the cranial endplate of the fourth thoracic vertebra (Th4) and expressed as total units of vertebral length. An enlarged LA is defined with an RLAD of above 1.8. The RLAD is a reproducible measurement and has a high sensitivity (94%) and specificity (97%), as well as a strong correlation with the LA:Ao (r = 0.82) [12,22]. These values are clearly higher than those of the “crossing lines method” of 70% sensitivity, 74% specificity, and only a moderate correlation with echocardiographic LA:Ao (r = 0.59). However, the strong correlation and high specificity of the RLAD method might be the result of patient selection because the included dogs had severe LA enlargement in this study [12,16]. Further studies are needed to evaluate if RLAD is also a reliable test for detecting less severe LA enlargement. Because RLAD was only tested by experienced observers, its performance is hard to assess in the hands of first-opinion veterinary practitioners or students. Also, bias may play a role in more experienced observers, as they might unconsciously use their subjective assessment when applying the RLAD method. Applying RLAD showed slight differences between observer groups with various types and levels of expertise [22]. Another study on RLAD showed that it could not be determined in 22% of the cases, probably due to difficulties in identifying the dorsal edge of the LA in dogs suffering from cardiogenic pulmonary edema [19]. 

The third described method, also published in 2018, is the vertebral LA size (VLAS) [13]. This method is obtained by drawing a straight line between the most ventral point of the tracheal bifurcation and the intersection between the cardiac silhouette and the dorsal border of the caudal vena cava on a lateral radiograph. Subsequently, this line is positioned along the vertebral bodies, starting at the cranial endplate of Th4, then the number of vertebral bodies is counted in a caudal direction, similarly to the VHS and RLAD calculations [13]. The result is expressed as the number of vertebral body lengths. An enlarged LA is defined as a VLAS > 2.3, and a VLAS ≥ 2.5 turned out to be the best cut off value to identify dogs with MMVD stage B2 [13,20]. Other studies showed too that VLAS could differentiate between stages B1 and B2 [17,18,22]. Though VLAS is a reproducible method [13,20,22], it might be slightly influenced by various levels of expertise [22]. The VLAS was only tested on dogs with enlarged LA due to MMVD and showed only a moderate positive correlation with echocardiography (r = 0.70) [13,22]. Though the sensitivity of VLAS was high (87%), the specificity was relatively low (67%) [22]. This high sensitivity might be caused by the fact that it was initially tested with a single experienced observer [13]. It was speculated that the lower specificity of the VLAS was due to the simplicity of the method [14]. However, the “crossing lines method” is even easier to perform than the VLAS but has a slightly higher specificity. In 7% of the cases VLAS could not be determined [19]. This percentage is clearly lower than that of RLAD, possibly because pulmonary infiltration affects VLAS less [22]. Therefore, VLAS is recommended to be used as a complementary rather than a confirmatory test [13]. Moreover, there is still some debate about whether VHS is a more clinically useful test than VLAS to identify LA enlargement in dogs with MMVD, as in this disease, both the left ventricle and LA dilate, leading to enlargement of the whole cardiac silhouette [16,20]. 

The fourth described method is the modified vertebral LA size (M-VLAS), reported in 2021 [14]. This method uses two lines instead of one. The first line is the same as used in the VLAS method. The second line starts at the most dorsal border of the LA, excluding the pulmonary veins, and is drawn perpendicularly with the first line [14]. Both lines are then drawn along the vertebrae starting again at the cranial endplate of Th4. The M-VLAS is defined as the sum of vertebral bodies corresponding to the length of both lines. An enlarged LA is defined as an M-VLAS > 3.4 [14]. The M-VLAS has a high sensitivity (93%) and specificity (93%), as well as a strong correlation with the LA:Ao (r = 0.77) [14]. Though the study suggested that the M-VLAS method worked better than the VLAS method, no significant difference was found [14]. The M-VLAS method does seem to be more accurate than the VHS [14]. Though VHS was not designed for measuring LA size, neither VLAS nor RLAD was superior to VHS in diagnosing LA enlargement. Moreover, it is hard to say whether the M-VLAS would be able to differentiate accurately between healthy dogs and dogs with cardiac disease due to the small number of healthy dogs (6 out of 70 cases) [14]. Another limitation of this study was that it was performed only on dogs with MMVD, and the method was applied by three experienced observers [14].

In contrast to the above-described methods, which intend to provide a quantitative assessment of the LA size, the “crossing lines method” aimed to differentiate normal LA from mildly to markedly enlarged LA. Application of the “crossing lines method” is faster, while it would still provide sufficient binary outcome (i.e., normal or big LA) for rapid clinical decision-making. The present study chose a LA:Ao of 1.7 as a cut-off value with an aim to detect even mild LA enlargement. However, a higher cut-off value, such as 2.0, might have resulted in a better performance of the test, especially if the aim is to differentiate cardiogenic pulmonary edema from pulmonary hypertension or a primary lung disease in dogs with respiratory distress. The present study enrolled dogs with various cardiac diseases, which might have influenced the performance of the “crossing lines method”, as the LA dilatation pattern might depend on the underlying cardiac disease. It is possible that the “crossing lines method” would perform better in a more homogenous sample regarding clinical presentation, breed, and type of underlying disease [23,24]. Whether chest conformation of various breeds and left versus right lateral recumbency had any influence on the performance of the “crossing lines methods” remains to be determined. 

There are a number of limitations of the present study. First, there was a bias when the observers were enrolled because they all participated on a voluntary basis. These participants were very motivated and had a natural interest in this subject, so they might have performed better than an average student in the first subjective interpretation round. The second limitation of the study is the suitability of echocardiographic LA:Ao as the gold standard because a linear measurement might not be accurate to determine the size of a complex three-dimensional structure, like the LA [3,25,26]. The third limitation is that the “crossing lines method” has not been compared to the other described methods (like RLAD, VLAS, and M-VLAS). The fourth limitation of the study is that no medical history and physical examination findings were made available to the observers. We chose not to offer these data because certain clinical findings, such as age, breed, murmur intensity, body condition score, respiratory rate, and effort, might have influenced the observers’ assessment and, in turn, the performance of the “crossing line method” [9]. Having said that, in a clinical setting, all these pieces of information would be integrated to reach a working diagnosis. The fifth limitation of the study is that certain cardiac diseases were represented in the study sample only by a single individual.

## 5. Conclusions

The present study was unable to show that the “crossing lines method” was superior to the subjective assessment of LA size on lateral radiographs in the hands of inexperienced observers. However, as the “crossing lines method” is less dependent on expertise, it might be used as a teaching tool and as an additional method to subjective or other standardized assessments of LA size on radiographs to increase accuracy. 

Future studies should evaluate the accuracy of the “crossing lines method” compared to other standardized methods, like (M-)VLAS and RLAD, preferably in an emergency setting or in first-opinion practices to establish whether the method helps to differentiate L-CHF from other causes of dyspnea.

## Figures and Tables

**Table 1:** Echocardiographic diagnoses of the 90 included dogs, whose radiographs were interpreted using two different methods.

Echocardiographic Diagnosis	Number of Dogs
Structurally normal heart	16
Myxomatous mitral valve degeneration	34
Dilated cardiomyopathy	19
Mitral valve dysplasia	6
Patent ductus arteriosus	5
Pulmonic stenosis	3
Aortic stenosis	2
Pericardial effusion	1
Pulmonary hypertension	1
Bradycardia-induced volume overload	2
Tachycardia-induced cardiomyopathy	1

**Table 2:** This table shows the percentage of true negatives, false negatives, true positives, and false positives when the four specialists in veterinary diagnostic imaging used subjective assessment for diagnosing left atrial enlargement on thoracic radiographs when the echocardiographic left atrial to aortic ratio (LA:Ao) was used as the gold standard. An agreement was reached in 81% of the cases.

		**LA:Ao**	
		**normal**	**enlarged**
Subjective assessment radiologists	**normal**	36%	6%
	**enlarged**	13%	45%

**Table 3:** **A**. This table shows the percentage of true negatives, false negatives, true positives, and false positives when the fourth-year students used their subjective assessments and the “crossing lines method” to determine left atrial size on lateral thoracic radiographs. The echocardiographic left atrial to aortic ratio (LA:Ao) was used as a reference. **B**. In this table, the mean scores of the fourth-year students’ assessments are shown when they used the subjective assessment and the “crossing lines method” to determine left atrial size on thoracic radiographs. To a case with a normal-sized left atrium, a 0 was allocated, and to a case with an enlarged left atrium, a 1; the mean score of the cases with a normal-sized left atrium would ideally be (as close as possible to) zero, and the mean score of the cases with an enlarged left atrium would ideally be (as close as possible to) 1, if there was a perfect agreement between the radiographic and the echocardiographic assessments. For the cases with a normal-sized left atrium, the subjective assessment of the left atrial size generated more correct diagnoses than the “crossing lines method” (*p* = 0.034).

**Fourth-Year Students**							
**A**							
		**LA:Ao**				**LA:Ao**	
		**normal**	**enlarged**			**normal**	**enlarged**
**Subjective assessment**	**normal**	36%	17%	**“Crossing lines method”**	**normal**	34%	17%
	**enlarged**	11%	36%		**enlarged**	13%	36%
**B**							
		**Subjective assessment**		**“Crossing lines method”**		***p*-value**	
Mean score of the cases with a normal atrium		0.231		0.275		*p* = 0.034	
Mean score of the cases with an enlarged atrium		0.675		0.674		*p* = 0.833	

**Table 4:** **A**. This table shows the percentage of true negatives, false negatives, true positives, and false positives when the sixth-year students used the subjective assessments and the “crossing lines method” to determine left atrial size on lateral thoracic radiographs. The left atrial size (normal or enlarged) was determined with the echocardiographic left atrial to aortic ratio (LA:Ao). **B**. In this table, the mean scores of the sixth-year students’ assessments are shown when they used the subjective assessment and the “crossing lines method” to determine left atrial size on lateral thoracic radiographs. The two methods showed no significant differences.

**Sixth-Year Students**							
**A**							
		**LA:Ao**				**LA:Ao**	
		**normal**	**enlarged**			**normal**	**enlarged**
**Subjective assessment**	**normal**	33%	14%	**“Crossing lines method”**	**normal**	35%	17%
	**enlarged**	14%	39%		**enlarged**	12%	36%
**B**							
		**Subjective assessment**		**“Crossing lines method”**		***p*** **-value**	
Mean score of the cases with a normal atrium		0.295		0.251		*p* = 0.361	
Mean score of the cases with an enlarged atrium		0.733		0.681		*p* = 0.274	

**Table 5:** **A**. This table shows the percentage of true negatives, false negatives, true positives, and false positives when the practicing veterinarians used the subjective assessments and the “crossing lines method” to determine left atrial size on lateral thoracic radiographs. The left atrial size (normal or enlarged) was determined by the echocardiographic left atrial to aortic ratio (LA:Ao). **B**. In this table, the means of the practicing veterinarians’ assessments are shown when they used the subjective assessments and the “crossing lines method” to determine left atrial size on lateral thoracic radiographs. The two methods showed no significant differences.

**Veterinarians**							
**A**							
		**LA:Ao**				**LA:Ao**	
		**normal**	**enlarged**			**normal**	**enlarged**
**Subjective assessment**	**normal**	36%	13%	**“Crossing lines method”**	**normal**	35%	14%
	**enlarged**	11%	41%		**enlarged**	12%	40%
**B**							
		**Subjective assessment**		**“Crossing lines method”**		***p*** **-value**	
Mean score of the cases with a normal atrium		0.235		0.256		*p* = 0.473	
Mean score of the cases with an enlarged atrium		0.765		0.681		*p* = 0.593	

**Table 6:** **A**. In this table, the accuracy, sensitivity, and specificity are shown for each observer group when using their subjective assessment to determine left atrial size on lateral thoracic radiographs. **B**. In this table, the accuracy, sensitivity, and specificity are shown for each observer group when using the “crossing lines method” for determining left atrial size on lateral thoracic radiographs.

	Fourth-Year Students	Sixth-Year Students	Veterinarians
**A: subjective assessment**			
Accuracy CI 95%	72% (70–74%)	72% (68–74%)	77% (74–80%)
Sensitivity CI 95%	67% (65–69%)	68% (65–71%)	76% (73–79%)
Specificity CI 95%	76% (74–78%)	75% (72–78%)	77% (74–80%)
**B: “crossing lines method”**			
Accuracy CI 95%	70% (68–72%)	71% (69–75%)	74% (71–77%)
Sensitivity CI 95%	67% (65–69%)	68% (65–71%)	74% (71–77%)
Specificity CI 95%	73 % (71–75%)	74% (71–77%)	74% (71–77%)

**Table 7:** **A**. In this table, the means of the fourth- and sixth-year students’ assessments are shown when the observers used their subjective assessments to determine left atrial size on lateral thoracic radiographs. For the cases with a normal atrium, the fourth-year students scored better. **B**. In this table, the means of the fourth-year students’ and practicing veterinarians’ assessments are shown when the observers used their subjective assessments to determine left atrial size on lateral thoracic radiographs. For the cases with an enlarged left atrium, the practicing veterinarians scored better. **C**. In this table, the means of the sixth-year students’ and practicing veterinarians’ assessments are shown when the observers used the subjective assessments to determine left atrial size on lateral thoracic radiographs. For the cases with a normal-sized left atrium, the practicing veterinarians scored better.

**A:** subjective assessment			
	**Fourth-Year Students**	**Sixth-Year Students**	***p*-Value**
Mean score of the cases with a normal atrium	0.231	0.295	*p* = 0.048
Mean score of the cases with an enlarged atrium	0.675	0.733	*p* = 0.079
**B:** subjective assessment			
	**Fourth-Year Students**	**Veterinarians**	***p*-Value**
Mean score of the cases with a normal atrium	0.231	0.235	*p* = 0.632
Mean score of the cases with an enlarged atrium	0.675	0.765	*p* = 0.044
**C:** subjective assessment			
	**Sixth-Year Students**	**Veterinarians**	***p*-Value**
Mean score of the cases with a normal atrium	0.295	0.235	*p* = 0.027
Mean score of the cases with an enlarged atrium	0.733	0.765	*p* = 0.172

**Table 8:** **A**. In this table, the means of the fourth- and sixth-year students’ assessments are shown when the observers used the “crossing lines method” to determine left atrial size on lateral thoracic radiographs. The two observer groups showed no significant differences. **B**. In this table, the means of the sixth-year students’ and practicing veterinarians’ assessments are shown when the observers used the “crossing lines method” to determine left atrial size on lateral thoracic radiographs. For the cases with an enlarged left atrium, the practicing veterinarians scored better. **C**. In this table, the means of the sixth-year students’ and the practicing veterinarians’ assessments are shown when the observers used the “crossing lines method” to determine left atrial size on lateral thoracic radiographs. No difference was found.

**A:** “crossing lines method”			
	**Fourth-Year Students**	**Sixth-Year Students**	***p*-Value**
Mean score of the cases with a normal atrium	0.275	0.251	*p* = 0.435
Mean score of the cases with an enlarged atrium	0.674	0.681	*p* = 0.709
**B:** “crossing lines method”			
	**Fourth-Year Students**	**Veterinarians**	***p*-Value**
Mean score of the cases with a normal atrium	0.275	0.256	*p* = 0.593
Mean score of the cases with an enlarged atrium	0.674	0.681	*p* = 0.045
**C:** “crossing lines method”			
	**Sixth-Year Students**	**Veterinarians**	***p*-Value**
Mean score of the cases with a normal atrium	0.251	0.256	*p* = 0.916
Mean score of the cases with an enlarged atrium	0.681	0.681	*p* = 0.072

## Data Availability

The data that support the findings of this study are available from the corresponding author upon reasonable request.

## Abbreviations

AUC = area under the curve; C.I. = confidence interval; LA = left atrium; LA:Ao = left atrial to aortic ratio; L-CHF = left-sided congestive heart failure; MMVD = myxomatous mitral valve disease; M-VLAS = modified vertebral left atrial size; PDA = patent ductus arteriosus; RLAD = radiographic left atrial dimension; ROC = receiver-operating characteristic curve; SE = standard error of mean; Th4 = the fourth thoracic vertebral body; VHS = vertebral heart scale; VLAS = vertebral left atrial size.
